# Pharmacy-based screening to detect persons at elevated risk of type 2 diabetes: a cost-utility analysis

**DOI:** 10.1186/s12913-021-06948-6

**Published:** 2021-09-05

**Authors:** Kari Jalkanen, Emma Aarnio, Piia Lavikainen, Jaana Lindström, Markku Peltonen, Tiina Laatikainen, Janne Martikainen

**Affiliations:** 1 Faculty of Health Sciences, School of Pharmacy, University of Eastern Finland, P.O. Box 1627, 70211 Kuopio, Finland; 2 Department of Public Health Solutions, Finnish Institute for Health and Welfare, P.O. Box 30, 00271 Helsinki, Finland; 3 Institute of Public Health and Clinical Nutrition, University of Eastern Finland, Faculty of Medicine, P.O. Box 1627, 70211 Kuopio, Finland; 4 Chronic Disease Epidemiology and Prevention Unit, Finnish Institute for Health and Welfare, Helsinki, Finland; 5 Joint Municipal Authority for North Karelia Health and Social Services (Siun Sote), Joensuu, Finland

**Keywords:** Type 2 diabetes, Pharmacy, Markov model, Cost effectiveness analysis

## Abstract

**Background:**

Early identification of people at elevated risk of type 2 diabetes (T2D) is an important step in preventing or delaying its onset. Pharmacies can serve as a significant channel to reach these people. This study aimed to assess the potential health economic impact of screening and recruitment services in pharmacies in referring people to preventive interventions.

**Methods:**

A decision analytic model was constructed to perform a cost-utility analysis of the expected national health economic consequences (in terms of costs and quality-adjusted life years, QALYs) of a hypothetical pharmacy-based service where people screened and recruited through pharmacies would participate in a digital lifestyle program. Cost-effectiveness was considered in terms of net monetary benefit (NMB). In addition, social return on investment (SROI) was calculated as the ratio of the intervention and recruitment costs and the net present value of expected savings. Payback time was the time taken to reach the break-even point in savings. In the base scenario, a 20-year time horizon was applied. Probabilistic and deterministic sensitivity analyses were applied to study robustness of the results.

**Results:**

In the base scenario, the expected savings from the pharmacy-based screening and recruitment among the reached target cohort were 255.3 m€ (95% CI − 185.2 m€ to 717.2 m€) in pharmacy visiting population meaning 1412€ (95% CI − 1024€ to 3967€) expected savings per person. Additionally, 7032 QALYs (95% CI − 1344 to 16,143) were gained on the population level. The intervention had an NMB of 3358€ (95% CI − 1397€ to 8431€) using a cost-effectiveness threshold of 50,000 €/QALY. The initial costs were 122.2 m€ with an SROI of 2.09€ (95% CI − 1.52€ to 5.88€). The expected payback time was 10 and 8 years for women and men, respectively. Results were most sensitive for changes in effectiveness of the intervention and selected discount rate.

**Conclusions:**

T2D screening and recruitment to prevention programs conducted via pharmacies was a dominant option providing both cost savings and QALY gains. The highest savings can be potentially reached by targeting recruitment at men at elevated risk of T2D.

**Supplementary Information:**

The online version contains supplementary material available at 10.1186/s12913-021-06948-6.

## Introduction

Diabetes is one of the most significant noncommunicable diseases with estimated 425 million adult patients in 2017 worldwide [1]. Its prevalence is increasing rapidly also in Finland, and currently around 350,000 Finns have type 2 diabetes (T2D) with some counties having up to 10% prevalence of T2D [2, 3]. In addition to the increasing number of T2D patients, a major challenge is the number of people at risk of T2D or with undiagnosed T2D. Currently, approximately 25% of the Finnish population have an elevated risk of developing T2D within the next 10 years [4].

T2D and its pre-stages have been shown to significantly increase the risk of complications and mortality [5, 6] as well as the related health and social care costs [7]. The incidence of T2D is strongly associated with low physical activity, unhealthy diet, and abdominal obesity, all of which can be influenced by supporting healthy lifestyles. The efficacy of lifestyle interventions in the prevention of T2D has been shown in several studies [8, 9], one of the first being the Finnish Diabetes Prevention Study (DPS) [10].

The incidence of T2D is the highest among people aged over 45 years [11]. One simple method for screening this population for T2D is to use a validated T2D risk assessment tool such as the Finnish Diabetes Risk Score (FINDRISC) test [12], which is a self-administered questionnaire used to estimate the 10-year risk of developing T2D.

Previous international studies have shown that pharmacies can act as an effective pathway to increase diabetes awareness, as well as to contact and identify people at elevated T2D risk [13, 14]. However, knowledge about the health economic impact of pharmacies in preventing T2D is limited [15]. The high number of customer contacts in pharmacies [16] provide a potential contact point for opportunistic risk screening for T2D with the use of a risk assessment tool.

Therefore, the aim of the present study was to conduct a cost-utility analysis to estimate the health economic consequences of a pharmacy-based screening and recruitment service conducted in the Finnish setting.

## Materials and methods

### Study setting

In Finland, the coverage of the pharmacy network is comprehensive, as there is at least one pharmacy in almost every municipality and, on average, one pharmacy per 6500 inhabitants [17]. The typical Finnish pharmacy delivers around 90,000 prescriptions per year with 46% delivering over 80,000 prescriptions in 2017 [18, 19]. Currently, around 60 million pharmacy visits occur in Finland yearly including purchases of both prescription and over-the-counter medications, and an estimated 2.1 million persons aged 30 to 79 years visit pharmacies yearly for their prescriptions [16, 20].

### Health economic modelling of pharmacy-based screening service

**Fig. 1:**
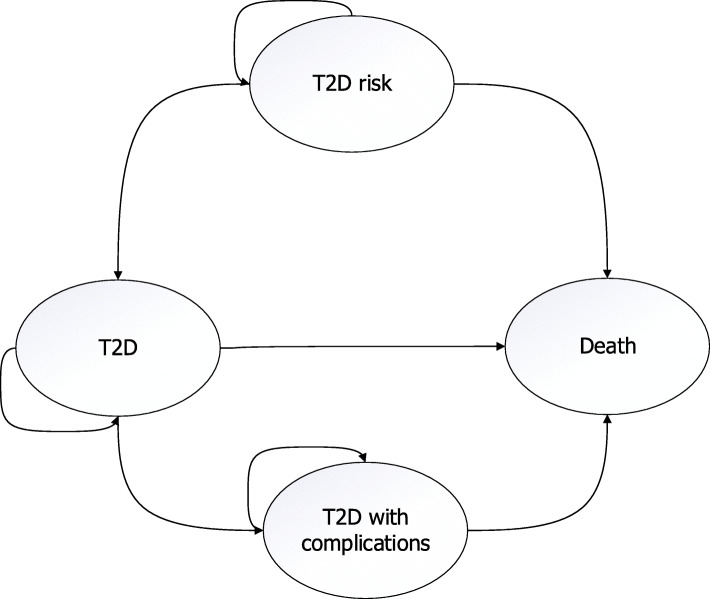
The simplified structure of the state transition model. The T2D with complications state stands for a person having any T2D-associated complication which are listed in (Additional file 1)

To estimate the national health economic consequences and cost-utility of a pharmacy-based screening service from a societal perspective, a health economic model was developed. The developed Markov-cohort model with annual cycles had four mutually exclusive health states (i.e., “T2D risk”, “T2D”, “T2D with complications” and “Death”) (Fig. 1). The applied time horizons were 10, 20, and 30 years. The parameters applied in the Markov model are shown in (Tables 1 and 2).

**Table 1:** Costs applied in the Markov model, their distributions and the values used to estimate the distributions. Costs before 2019 have been discounted to the latest values

Parameter	Value (€) (variation)	Distribution	Distribution values used in PSA (€) Mean (SE)	Source
Cost of recruitment*	24.5€ (18.4–30.6)	Gamma	24.5 (3)	Based on own results
Cost of intervention*	650 € (488–813)	Gamma	650 (83)	[21]
Additional health care costs of T2D excluding basic health care*	3315 € (2486–4144)	Gamma	3315 (423)	[22]
Cost of T2D complications*	4401€ (3301–5501)	Gamma	4401 (561)	[23]
Costs from productivity losses due to T2D ^a*^	7632€ (5724–9540)	Gamma	7632 (974)	[24]
Additional T2D health care costs for basic health care	551 (SD 575) for men 533 (SD 635) for women	Gamma	Men = 551 (9.53) Women = 533 (9.82)	Based on own results
Additional medication costs of T2D*	584 (438–730)	Gamma	584 (74)	[25]

^a^ for persons under 65 years old *For variables without available confidence interval, a variation of ±25% has been used as an estimate. SE was calculated separately for additional T2D health care costs for basic health care. In other these cases, SE has been calculated as:

$$ SE=\frac{Mean\ast 1.25- Mean\ast 0.75}{2\ast 1.96} $$

**Table 2:** Parameters applied in the Markov model, their distributions and the values used to estimate the distributions

Parameter	Value (variation)	Distribution	Distribution values used in PSA Mean (SE)		Source
**Hazard ratios**					
Effect of intervention (95% CI)	0.74 (0.53–1.03)	Lognormal	0.74 (0.17)		[26]
T2D-specific mortality risk (95% CI)	HR 2.47 (2.42–3.06) (women)	Lognormal	2.47 (0.04)		[27]
	HR 1.93 (1.79–2.07) (men)		1.93 (0.05)		
Mortality risk associated with T2D with complications	HR 2.36 (1.70–3.29)	Lognormal	2.36 (0.41)		[28]
All-cause mortality^a^	Based on age and gender	–	–		[29]
**Utilities**					
Baseline utilities (EQ-5D-3L)	**Women** **(Age, Utility, SE)**	Beta	**Alpha** **(Age, value)** **Women**	**Beta** **(Age, value)** **Women**	[30]
	30–44 0.906 (0.003)		30–44 8573	30–44, 889	
	45–54 0.865 (0.005)		45–54 4040	45–54, 631	
	55–64 0.810 (0.006)		55–64 3463	55–64, 812	
	65+ 0.770 (0.008)		65+ 2130	65+ 636	
	**Men** **(Age, Utility, SE)**		**Men**	**Men**	
	30–44 0.917 (0.003)		30–44 7755	30–44, 702	
	45–54 0.876 (0.005)		45–54 3806	45–54, 539	
	55–64 0.821 (0.006)		55–64 3351	55–64, 731	
	65+ 0.781 (0.008)		65+ 2087	65+ 585	
Disutility of T2D (EQ-5D-3L) (SE)	0.041 (0.012)	Beta	**Alpha** 11.19	**Beta** 261.9	[31]
Weighted disutility of T2D complications (EQ-5D-3L)^b^	0.119 (0.089–0.149)	Beta	0.119 (0.015)		Disutility values of individual complications [32–36] Proportion of complications [37]

^a^All-cause mortality data is shown in additional file 7

^b^For variables without available confidence interval, a variation of ±25% has been used as an estimate. In these cases, SE has been calculated as:

$$ SE=\frac{Mean\ast 1.25- Mean\ast 0.75}{2\ast 1.96} $$

### Population

The size of the modelled population cohorts conditional on gender were adjusted to correspond with the Finnish population [38] visiting community pharmacies annually [16], and having no T2D at baseline [4]. The distribution of the baseline risk for T2D (assessed with FINDRISC scores) conditional on age and gender, was defined based on the baseline characteristics of the Stop Diabetes (StopDia) study [39]. Based on these available data, a hypothetical baseline cohort of men and women aged 30 to 79 years at elevated T2D risk was constructed by combining the gender-specific cohorts. For an example of the applied approach to define this reached cohort, see (Additional File 2).

### Transition probabilities

The annual probabilities of T2D incidence conditional on age, gender, and baseline FINDRISC scores, (Additional File 3), were obtained from post-hoc survival analyses of the National FINRISK study [40] follow-up datasets enriched with data from the national medicine reimbursement register maintained by the Social Insurance Institution. Based on these data, a Weibull survival regression model was fitted to estimate the relationships between baseline age, gender, baseline FINDRISC scores, and the incidence of T2D during, on average, a 10-year follow-up to enable the extrapolation of transition probabilities over the actual follow-up time. Akaike information criteria and Bayesian information criteria were used to select the best model, the Weibull model having the lowest values and thus chosen for the final model. The values used for selection are shown in (Additional file 4). In addition, the visual inspection of the parametric survival curves supported this selection. The model fit of the Weibull regression was estimated using Wald-Chi test with a value of χ2(6) = 143.80, *p* < 0.001. *P*-values less than 0.05 were used to indicate statistical significance. The coefficients of the Weibull regression for incidence of T2D are shown in (Additional file 5).

In people with newly diagnosed T2D, the age- and gender-specific annual probability of T2D-related complications (i.e., the transition from the “T2D” state to the “T2D with complications” state) was estimated based on real-world electronic health record data of patients (*n* = 1151) with newly diagnosed T2D between 2011 and 2012 and living in the county of North Karelia. The data was available until December 2019 with the longest follow-up duration of 9.0 years. A Weibull regression model was fitted to estimate the annual rate of complications based on gender and age. The same selection criteria were used as with the first regression model. The model fit of the regression was estimated using Wald-Chi test with a value of χ2(2) = 95.87, *p* < 0.001. The coefficients of the Weibull regression for incidence of T2D complications are shown in (Additional file 6). The FINDRISC groups (0–6, 7–11, 12–14, 15–19 and 20–26 points) serve as the regression coefficients of the model. The micro- and macrovascular complications which were regarded as T2D-related were stroke, neuropathy, and foot, kidney, and cardiovascular complications. For more accurate details, see (Additional File 1).

All-cause mortality rates for men and women in the population cohorts were obtained from the national life-tables [29]. These are shown in (Additional file 7). Increased risk of death due to T2D was considered by applying mortality risk ratios obtained from a previously published study (Table 2). In addition, the “T2D with complications” state was expected to be associated with a further increased risk of death [28].

### Reach of recruitment

In the base scenario, the proportions of detected cases with elevated T2D risk among pharmacy visitors were estimated from the recruitment phase data of the StopDia study [41]. In the StopDia study, pharmacies were one of the recruitment channels that were used to contact, identify, and recruit people over 18 years old at T2D risk to take part in the lifestyle intervention. People with FINDRISC scores ≥12, history of pregnancy diabetes or elevated blood glucose levels were considered as having elevated risk and, therefore, eligible to participate in the StopDia study. During the recruitment phase, pharmacies had printed FINDRISC forms available for customers. Pharmacy staff received training regarding the identification of T2D risk factors and recruiting participants. People identified as having elevated T2D risk in the pharmacies were counselled to proceed to fill in the electronic screening tool on the StopDia website and encouraged to take part in the StopDia study. In total of 36 pharmacies from study areas participated in the recruitment phase of the StopDia study.

In StopDia, a total of 6705 FINDRISC forms were handed out in pharmacies, and this resulted in 662 persons (9.9%) completing the FINDRISC online. To scale-up these results nationwide, the number of pharmacy visitors was estimated from data by the Finnish Medicines Agency [16], varying from 30 to 90% of the whole population based on age and gender with an average of 61%, see (Additional File 8). Next, the size of the cohorts applied in the model was estimated to be the number of people without T2D visiting pharmacies each year multiplied by the reach of the recruitment (9.9%). By scaling-up these results into the whole Finnish population visiting pharmacies, the number of people possibly reachable via a nationwide service was expected to be 180,774 persons (100,399 women and 80,375 men) annually (i.e., the reached population).

### Effectiveness of lifestyle intervention

The effect of a lifestyle intervention was modelled via weight loss (kg) since it has been shown to be significantly associated with the incidence of T2D [42]. As a comparator we used a scenario without any lifestyle intervention (current practice in Finland). In the present study, the duration of the lifestyle intervention was assumed to be one year and, on average, it was assumed to lead to a modest 2.5–4.9% weight reduction (as compared with the baseline) during a year [43–45]. The association between the expected weight loss and the long-term incidence of T2D was estimated based on a post-hoc analysis of the Finnish DPS follow-up data [42]. The number of persons in the follow-up was 9512 and the number of T2D cases was 251. The conducted post-hoc analysis showed that a weight loss of 2.5–4.9% during the first year of intervention results in a 26% (HR 0.74, 95% CI 0.53–1.03) risk reduction, on average, in the incidence of T2D over a 15-year follow-up period adjusted for age, gender, BMI and fasting glucose. After 15 years, the effect was conservatively assumed to end immediately in the model without any catch-up period.

### Cost and utility data

The cost of the pharmacy-based screening and recruitment service was estimated based on the assumed value of the working time of pharmacists: it was estimated that counseling per participant would take 5 min (1.93€). Other costs included leaflets (0.5€ a piece) distributed to customers. Considering the expected 9.9% rate of reaching the customers, this cost totals to 25.4€ per eligible pharmacy visitor (i.e., recruited and people either participating in the intervention or not).

The cost of the lifestyle intervention was assumed equal with the cost of a previous Finnish digital obesity prevention lifestyle intervention (i.e., 650€ per participant) [21]. The costs were assumed to occur during the first year of the intervention. Together with the costs of screening and recruitment, the total costs per person in the intervention was estimated to be, on average, 675€. A discount rate of 3% was applied in the analysis. The costs applied in the model are shown in Table 1.

The societal perspective, allowing the inclusion of both direct health care costs and costs from productivity loss due to T2D, as well as the costs of screening, recruitment, and the intervention, was applied in the analysis. The direct annual additional monitoring and complications costs of T2D were obtained from a previous national register study [24]. Since this national register study did not include additional primary care costs associated with T2D, they were estimated based on the same real-world electronic health record data from North Karelia, which was also applied to estimate the annual incidence of T2D-related complications. Costs from productivity losses due to T2D consisted of premature retirement under the age of 65, death, and sick leaves associated with T2D [24].

The utility values used in the model were based on the EQ-5D-3L instrument [46]. The base utility and disutility values used are shown in (Table 2) and are based on studies performed among the Finnish population [30, 31]. The disutility of complications associated with T2D were calculated by using the proportion of the complications used in a previous study performed on the national level [37] and by weighing them with the disutility associated with each complication [32]. The standard error for the resulting disutility (0.119) was calculated by estimating a variation of ±25%.

### Presentation of the results

All costs and quality-adjusted life years (QALYs) were cumulatively summarized over the applied time horizon to estimate the total additional costs of T2D with and without the pharmacy-based service and lifestyle intervention. In the base case a time horizon of 20 years was applied. Results were presented as the total expected savings in the reached population as well as savings per individual person.

The concept of net monetary benefit (NMB) was used to assess the cost-effectiveness of the pharmacy-based screening and recruitment [47]. The NMB was calculated using the formula NMB = (ΔQALY*WTP)- Δcosts, where WTP is the willingness to pay for a unit of benefit. In addition, the social return on investment (SROI) was estimated for the pharmacy-based screening and recruitment. This value can be used to estimate the attributable savings [48]. In this study, SROI was estimated by dividing the discounted expected savings by the cost of the pharmacy-based screening and recruitment. The concept of payback time was used to estimate the time (in years) how long it would take for the pharmacy-based service to reach a break-even point in the form of attained savings [49].

### Sensitivity analyses

In the base scenario, probabilistic sensitivity analysis (PSA) was used to determine the 95% confidence intervals (CIs) for the expected savings and QALY gains. Parameter uncertainty was handled by determining probability distributions for all relevant parameters and then conducting PSA with 1000 random iteration rounds [50]. Suitable parameter distributions for the PSA were selected based on previous literature [51]. The tested parameters are shown in (Tables 1 and 2). The correlation structure between the Weibull regression coefficients was also considered in the PSA and the regression coefficients were assumed to be normally distributed. For the correlation matrix, see (Additional File 9).

In addition, one-way sensitivity analyses were conducted to test the robustness of model assumptions by adjusting the values of the following variables by ±25%: additional health care costs of T2D, costs from productivity losses due to T2D, intervention cost, the costs associated with T2D complications, and the costs of screening and recruitment. The HR of the intervention was varied between its 95% CI (0.53 to 1.03). Furthermore, the discount rate was varied from 0 to 5% for both costs and QALYs.

## Results

### Base scenario results

**Fig. 2:**
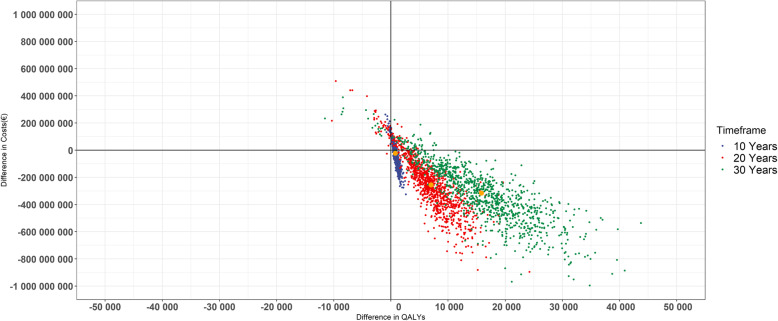
Results of the PSA with 1000 random iterations on the cost-effectiveness plane showing the expected savings (€) and QALY gains in the whole reached population (*n* = 180,774). The results of the intervention have been shown over a timeframe of 10, 20, and 30 years. The point estimate is presented with a yellow color dot

Considering both direct health care costs and costs from productivity losses due to T2D, the total health care system savings for the pharmacy-based screening and recruitment among the reached population of 180,774 persons were 255.3 m€ (95% CI − 185.2 m€ to 717.2 m€) (Fig. 2) corresponding with 1412€ (95% CI − 1024€ to 3967€) expected savings per person with men receiving larger benefits from the intervention than women, 1889€ (95% CI − 1053€ to 4846€) and 1030€ (95% CI - 1001€ to 3264€), respectively (Table 3). In the base case 3.8% of scenarios resulted in cost and QALY losses, 5% of the scenarios resulted in cost losses but QALY gains, 0.1% of the scenarios resulted in cost saving but QALY losses and 91.2% of the scenarios resulted in both cost savings and QALY gains. The corresponding SROI was 2.09€ (95% CI − 1.52€ to 5.88€). When considering both direct health care and costs from productivity losses due to T2D, the expected payback time was 10 and 8 years for women and men, respectively.

**Table 3:** The expected savings and QALYs gained on an individual and population level among 100,399 women and 80,375 men in non-T2D pharmacy visiting population. Comparator for the intervention was current practice

	10-year time period		20-year time period		30-year time period	
	Women	Men	Women	Men	Women	Men
**Expected savings potential per person € (95% CI)**	15 (− 799 to 945)	268 (− 799 to 1520)	1031 (− 1002 to 3264)	1889 (− 1053 to 4846)	1457 (− 1085 to 4024)	2115 (− 816 to 5239)
**Expected savings potential per person Only direct costs € (95% CI)**	− 410 (− 738 to − 39)	− 228 (− 738 to 343)	625 (− 1012 to 2472)	1264 (− 1105 to 3601)	1044 (− 967 to 3107)	1526 (− 1133 to 3920)
**Expected gained QALYs per person (95% CI)**	0.0031 (−0.0004 to 0.0074)	0.0055 (− 0.0008 to 0.0133)	0.0270 (− 0.0054 to 0.0623)	0.0538 (− 0.010 to 0.1230)	0.0684 (− 0.0141 to 0.1512)	0.0877 (− 0.0102 to 0.1904)
**Expected savings potential, population m€ (95% CI)**	1.5 (−80.2 to 94.9)	21.5 (−64.3 to 122.1)	103.5 (− 100.5 to 327.7)	151.8 (− 84.7 to 389.5)	146.2 (− 108.9 to 404.0)	170.0 (− 65.6 to 421.1)
**Expected savings potential, population Only direct costs m€ (95% CI)**	− 41.2 (− 74.1 to − 3.9)	−18.3 (− 59.4 to 27.6)	62.7 (− 101.6 to 248.2)	101.6 (− 88.8 to 289.4)	104.9 (− 97.1 to 312.0)	122.9 (− 91.1 to 315.0)
**Expected gained QALYs, population (95% CI)**	309 (−43 to 738)	445 (−61 to 1068)	2712 (− 540 to 6257)	4320 (− 804 to 9886)	6869 (− 1418 to 15,178)	8979 (− 435 to 19,239)
**Net monetary benefit, cost-effectiveness threshold of 50,000 €/QALY (€) (95% CI)**	170 (− 819 to 1315)	543 (− 839 to 2185)	2381 (− 1272 to 6379)	4579 (− 1553 to 10,996)	4877 (− 1790 to 11,584)	6500 (− 1326 to 14,759)

*CI* Confidence interval, *QALY* Quality-adjusted life year

The discounted savings, when taking only direct health care costs into account (i.e., without costs from productivity losses due to T2D), were 164.3 m€ (95% CI − 190.4 m€ to 538.0 m€) in the reached population and 909€ (95% CI - 1053€ to 2976€) on an individual level (Table 2). The SROI was 1.35€ (95% CI − 1.56€ to 4.41€) and expected payback time 14 and 11 years for women and men, respectively.

Furthermore, 7032 additional QALYs (95% CI − 1344 to 16,143) were gained in the base scenario (Table 3). When considering the cost-effectiveness of the pharmacy-based service and the intervention, recruitment resulting in participating in the intervention was a dominant option (i.e., less costly and more effective) in the base scenario. In the 20-year scenario, the NMB estimate for men was 4579 € (95% CI − 1553 to 10,996 €) and for women 2381 € (95% CI − 1272 to 6379 €) and the gender- weighted estimate was 3358€ (95% CI − 1397€ to 8431€) using a cost-effectiveness threshold of 50,000 €/QALY. The uncertainty associated with the results increased with the timeframe, the uncertainty of QALYs increasing relatively more than the uncertainty of costs.

### Results of one-way sensitivity analyses

**Fig. 3:**
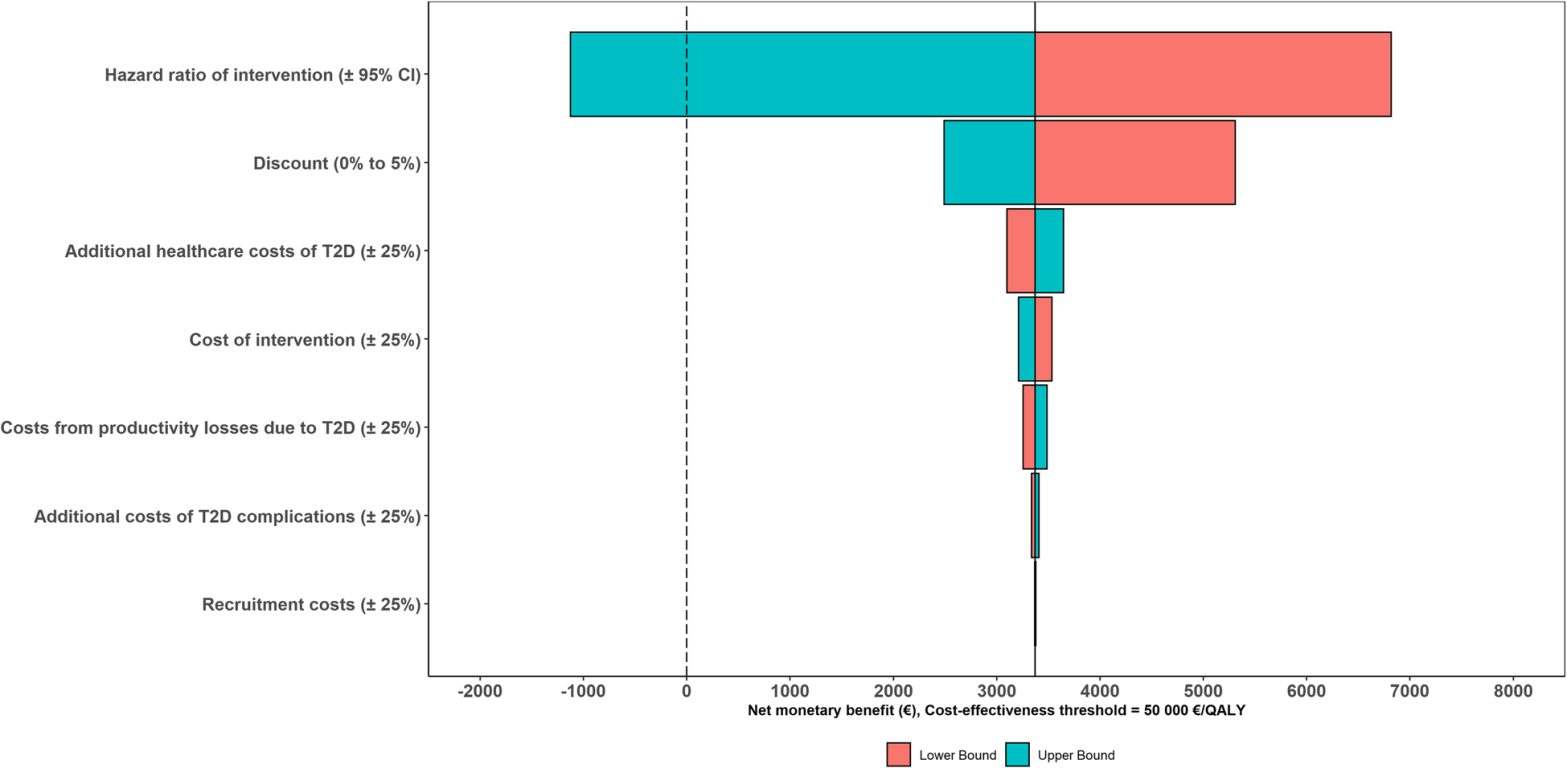
Expected net monetary benefit (€) of the different one-way scenarios for the intervention in the base scenario with 20-year timeframe using a cost-effectiveness threshold of 50,000 €/QALY

Based on one-way sensitivity analyses regarding NMB, the effectiveness of the lifestyle intervention had the largest effect on the results, followed by discount rate and the additional health care costs of T2D. Variating the HR of the intervention within its 95% CI (0.53 to 1.03) resulted in variation of NMB from − 1124€ to 6818€ (Fig. 3). Variating the discount rate from 0 to 5% resulted the NMB changing from 2419€ to 5310€ and variating the additional health care costs of T2D by 25% changed the NMB from 3098€ to 3646€. Variating the recruitment costs by 25% only minimally affected NMB (3378€ to 3366€).

## Discussion

According to our study findings, the pharmacy-based screening and recruitment service could potentially lead to net cost savings and increased number of QALYs at the population level. The NMB of the intervention was positive using a cost-effectiveness threshold of 50,000 €/QALY. A large proportion of the expected savings could be expected to come from smaller costs due to productivity losses of people aged under the common Finnish retirement age of 65 years.

In the present study, the rate of recruitment was based on the results of the national StopDia study, reflecting real-world circumstances in the pharmacy-based screening and recruitment, where 9.9% of visitors at T2D risk were reached. If the amount of people reached were to increase from 9.9% to, for example, 12.4%, the achievable savings would increase regardless of higher recruitment costs. The cost of reaching people at T2D risk in pharmacies has been previously estimated to be, on average, 84€ per participant in a study conducted in Sweden [52] and 29£ (33.06€) in a study from the UK [15]. Our costs were estimated to be lower due to a less intensive recruitment procedure without blood glucose testing.

In our model, a 26% reduction in T2D risk (over a period of 15 years) was applied as the effectiveness of the digital intervention. This risk reduction was based on a weight loss of 2.5–4.9% during the first year. Digital programs have shown to result in weight loss of 3 to 7.5% [43, 44, 53–55], and intensive lifestyle interventions have been shown to reduce the risk of T2D by 33–43% [9, 42, 56]. Compared with these results, our estimate of effectiveness can be considered conservative. The portion of recruited pharmacy visitors was estimated to be higher, 9.9%, than in a study conducted in Switzerland, 2.4% [14], but in that study the recruitment period was limited to 5 weeks whereas in our study the recruitment is assumed to continue for a year. Pharmacies may have a very important role in screening for T2D as the population that visits pharmacies tends to be middle-aged or older and thus at a higher risk of T2D [16]. People who visit pharmacies may be more receptive to advice and they can receive it regularly when they collect their prescriptions [14].

### Strengths and limitations of the study

We applied the FINDRISC score data collected in the StopDia study to characterize the baseline risk of T2D, which can be considered to characterize the reached population more accurately than the corresponding national averages. Second, we used national registers and real-world datasets to estimate the incidence of T2D and micro- and macrovascular complications in persons with newly diagnosed T2D, in addition to primary care costs associated with T2D.

Naturally, there are also some limitations in our study. First, our model used data from both Finland and other countries. Data on T2D mortality from other countries may not accurately represent the situation in Finland. Second, due to the StopDia study protocol, the recruitment was only focused on persons under the age of 70, leaving out an age group at high risk of T2D.

## Conclusions

Our results indicate that pharmacies can serve as a cost saving channel to reach people at elevated risk of T2D. The prevention program was dominant in the 20-year scenario as both savings and QALY gains were achieved. Highest savings can be gained by targeting the screening and recruitment at men who are in elevated risk of T2D. A large part of the savings came from lower costs from productivity losses due to T2D. On a population level, higher savings in costs and more QALYs may be attained if pharmacies are able to reach a larger part of the population at risk of T2D.

## Supplementary Information

**Additional file 1.** The complications considered to be T2D-related in the Weibull regression model. Table showing the ICD-10 codes and NOMESCO codes of the T2D complications.

**Additional file 2.** Population used in the Markov model showing the steps used to estimate the size of the reached population cohort. A graph showing how the population cohort was estimated.

**Additional file 3 **The FINDRISC- score distribution for the people who filled a baseline questionnaire and were eligible to participate in the StopDia study (*n* = 5882). Table containing FINDRISC score distributions.

**Additional file 4.** The Akaike Information Criteria and Bayesian Information Criteria values used to select best fitting model. Table containing the statistical criteria used in selecting the model used in the study.

**Additional file 5.** Coefficients of the Weibull regression for incidence of T2D. Table showing the coefficients of the Weibull regression for incidence of T2D.

**Additional file 6.** Coefficients of the Weibull regression for incidence of T2D-related complications. Table showing the coefficients of the Weibull regression for incidence of T2D-related complications.

**Additional file 7.** Age and gender- specific all-cause mortality data in Finland. Table showing the all-cause mortality data according to Statistics Finland.

**Additional file 8.** Baseline characteristics of the cohorts used in the Markov model. Table showing the characteristics of the population cohorts by gender.

**Additional file 9.** The correlations between the Weibull regression coefficients. Table showing the correlations between the coefficients for the Weibull to estimate the risk of T2D and the rate of complications.

## Data Availability

The data that support the findings of this study are available from Joint Municipal Authority for North Karelia Social and Health Services and the StopDia project, but restrictions apply to the availability of these data, which were used under license for the current study, and so are not publicly available. Data are however available from the authors upon reasonable request and with permission of Joint Municipal Authority for North Karelia Social and Health Services and the StopDia project.

## Abbreviations

CI: Confidence interval
DPS: Diabetes Prevention Study
QALY: Quality-adjusted life year
NMB: Net monetary benefit
PSA: Probabilistic sensitivity analysis
SROI: Social return of investment
WTP: Willingness to pay
T2D: Type 2 diabetes
