# Supplementary material for: Pharmacy-based screening to detect persons at elevated risk of type 2 diabetes: a cost-utility analysis
Source: BMC Health Serv Res. 2021 Sep 5;21:916. doi: 10.1186/s12913-021-06948-6 (PMC8418722; doi:10.1186/s12913-021-06948-6)
Supplement: Supplementary file 1 — Additional file 1. The complications considered to be T2D-related in the Weibull regression model. Table showing the ICD-10 codes and NOMESCO codes of the T2D complications. [file 12913_2021_6948_MOESM1_ESM.docx]

**Additional file 1**. The complications considered to be T2D-related in the Weibull regression model

| **Complication** | **ICD-10** |
| --- | --- |
| **Eye complications** |  |
| Retinopathy or other diabetic eye complication, | E11.3, H28.0, H36 (excluding H36.8), H40.5, H42.0, H43.1, H45.0, |
| **Renal complications** |  |
| Renal insufficiency | E11.2, N08.3, N18 |
| End-stage renal disease (with or without dialysis) | Z49, Z94.0 |
| **Neuropathic complications** |  |
| Amputation of lower extremities | NOMESCO: NFQ10, NFQ20, NGQ10, NGQ20, NHQ10, NHQ20, NFQ48, NGQ48, NHQ30, NHQ40, NHQ60 |
| Diabetic neuropathy, Charcot foot or other diabetic foot complication | E11.4, E11.5, E11.6, G59.0, G63.2, G73.0, G99.0, I70.2, I73.9, I79.2, L97, M14.2, M14.6, N48.4 |
| **Cardiovascular complications** |  |
| Angina pectoris | I20.0 |
| Chronic heart failure | I11.0, I13.0, I13.2, I50 |
| Incident myocardial infarction/cardiac arrest | I21, I46 |
| Other ischemic heart disease | I22–I25 |
| **Cerebrovascular complications** |  |
| Ischemic stroke | I63–I66 (excluding I63.6) |
