# Supplementary material for: Pharmacy-based screening to detect persons at elevated risk of type 2 diabetes: a cost-utility analysis
Source: BMC Health Serv Res. 2021 Sep 5;21:916. doi: 10.1186/s12913-021-06948-6 (PMC8418722; doi:10.1186/s12913-021-06948-6)

**Additional file 2.** Population used in the Markov model showing the steps used to estimate the size of the target population cohort.

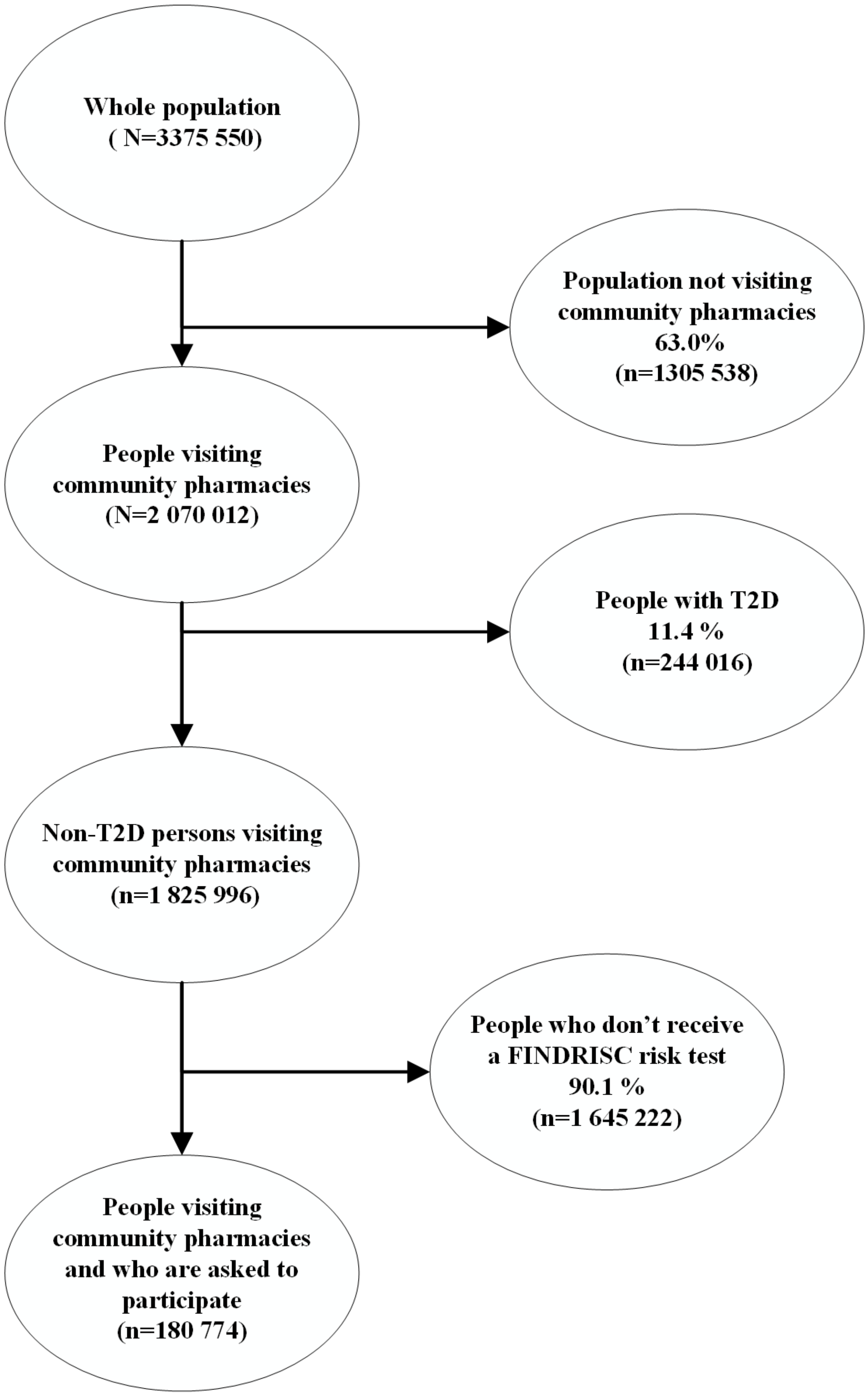

Supplement: Supplementary file 2 — Additional file 2. Population used in the Markov model showing the steps used to estimate the size of the reached population cohort. A graph showing how the population cohort was estimated. [file 12913_2021_6948_MOESM2_ESM.pdf]
