# Supplementary material for: Pharmacy-based screening to detect persons at elevated risk of type 2 diabetes: a cost-utility analysis
Source: BMC Health Serv Res. 2021 Sep 5;21:916. doi: 10.1186/s12913-021-06948-6 (PMC8418722; doi:10.1186/s12913-021-06948-6)
Supplement: Supplementary file 3 — Additional file 3 The FINDRISC- score distribution for the people who filled a baseline questionnaire and were eligible to participate in the StopDia study (n = 5882). Table containing FINDRISC score distributions. [file 12913_2021_6948_MOESM3_ESM.docx]

**Additional file 3**. The FINDRISC- score distribution for the people who filled a baseline questionnaire and were eligible to participate in the StopDia study (n=5 882).

| **Gender** | **Age** | **FINDRISC** | **FINDRISC** | **FINDRISC** | **FINDRISC** | **FINDRISC** |
| --- | --- | --- | --- | --- | --- | --- |
|  |  | **0–6** | **7–11** | **12–14** | **15–19** | **20–26** |
| **Men** | **30 to 39** | 0 | 0.01 | 0.59 | 0.35 | 0.03 |
| **Men** | **40 to 49** | 0 | 0.02 | 0.43 | 0.45 | 0.07 |
| **Men** | **50 to 59** | 0 | 0 | 0.32 | 0.46 | 0.2 |
| **Men** | **60 to 69** | 0 | 0.01 | 0.3 | 0.47 | 0.21 |
| **Men** | **70 to 79** | 0 | 0 | 0.16 | 0.68 | 0.16 |
| **Women** | **30 to 39** | 0 | 0.07 | 0.46 | 0.40 | 0.05 |
| **Women** | **40 to 49** | 0 | 0.03 | 0.36 | 0.49 | 0.1 |
| **Women** | **50 to 59** | 0 | 0.01 | 0.28 | 0.48 | 0.21 |
| **Women** | **60 to 69** | 0 | 0 | 0.27 | 0.46 | 0.25 |
| **Women** | **70 to 79** | 0 | 0 | 0.27 | 0.46 | 0.25 |
