# Supplementary material for: Pharmacy-based screening to detect persons at elevated risk of type 2 diabetes: a cost-utility analysis
Source: BMC Health Serv Res. 2021 Sep 5;21:916. doi: 10.1186/s12913-021-06948-6 (PMC8418722; doi:10.1186/s12913-021-06948-6)
Supplement: Supplementary file 4 — Additional file 4. The Akaike Information Criteria and Bayesian Information Criteria values used to select best fitting model. Table containing the statistical criteria used in selecting the model used in the study. [file 12913_2021_6948_MOESM4_ESM.docx]

**Additional file 4**. The Akaike Information Criteria and Bayesian Information Criteria values used to select best fitting model.

| **Model** | **Observations** | **Log-likelihood (observed)** | **Log-likelihood (model)** | **df** | **AIC** | **BIC** |
| --- | --- | --- | --- | --- | --- | --- |
| Weibull | 9 512 | -1 160.698 | -1 020.396 | 8 | 2 056.792 | 2 114.075 |
| Exponential | 9 512 | -1 238.977 | -1 106.648 | 7 | 2 227.296 | 2 277.419 |
| Gompertz | 9 512 | -1 173.391 | -1 032.211 | 8 | 2 080.423 | 2 137.705 |
| Lognormal | 9 512 | -1 162.251 | -1 022.406 | 8 | 2 060.812 | 2 118.095 |
| Generalized Gamma | 9 512 | -1 160.302 | -1 019.661 | 9 | 2 057.321 | 2 121.764 |
