# Supplementary material for: Pharmacy-based screening to detect persons at elevated risk of type 2 diabetes: a cost-utility analysis
Source: BMC Health Serv Res. 2021 Sep 5;21:916. doi: 10.1186/s12913-021-06948-6 (PMC8418722; doi:10.1186/s12913-021-06948-6)
Supplement: Supplementary file 5 — Additional file 5. Coefficients of the Weibull regression for incidence of T2D. Table showing the coefficients of the Weibull regression for incidence of T2D. [file 12913_2021_6948_MOESM5_ESM.docx]

**Additional file 5**. Coefficients of the Weibull regression for incidence of T2D.

| **Parameter** | **Value (variation)** | **p-value** | **Distribution** | **Distribution values used in PSA**  **Mean (SE)** |
| --- | --- | --- | --- | --- |
| **Weibull regression coefficients, risk of T2D**  **(95 % CI)** |  |  |  |  |
| Weibull Gamma | 2.329 (2.101 to 2.582) | >0.001 | Normal | 2.329 (0.122) |
| Gender Coefficient | -0.256 (-0.370 to -0.150) | >0.001 | Normal | -0.256 (0.256) |
| Age Coefficient | -0.002 (-0.091 to 0.005) | 0.532 | Normal | -0.002 (0.0016) |
| Constant | 4.668 (4.158 to 5.185) | >0.001 | Normal | 4.668 (1.199) |
| FINDRISC 0-6 | 0.000 (0.000 to 0.000) | Reference | Normal | 0.000 (0.000) |
| FINDRISC 7-11 | -0.486 (-0.779 to -0.192) | >0.001 | Normal | -0.486 (0.691) |
| FINDRISC 12-14 | -0.908 (-1.200 to -0.615) | >0.001 | Normal | -0.908 (0.687) |
| FINDRISC 15-19 | -1.284 (-1.569 to -0.998) | >0.001 | Normal | -1.284 (0.672) |
| FINDRISC 20+ | -1.658 (-1.964 to -1.351) | >0.001 | Normal | -1.658 (0.719) |

The FINDRISC score groups (0-6, 7-11, 12-14, 15-19 and 20-26) are beta coefficients of the regression.
