# Supplementary material for: Pharmacy-based screening to detect persons at elevated risk of type 2 diabetes: a cost-utility analysis
Source: BMC Health Serv Res. 2021 Sep 5;21:916. doi: 10.1186/s12913-021-06948-6 (PMC8418722; doi:10.1186/s12913-021-06948-6)
Supplement: Supplementary file 6 — Additional file 6. Coefficients of the Weibull regression for incidence of T2D-related complications. Table showing the coefficients of the Weibull regression for incidence of T2D-related complications. [file 12913_2021_6948_MOESM6_ESM.docx]

**Additional file 6**. Weibull regression coefficients for the incidence of T2D-related complications.

| **Parameter** | **Value (variation)** | **p-value** | **Distribution** | **Distribution values used in PSA**  **Mean (SE)** |
| --- | --- | --- | --- | --- |
| **Weibull regression coefficients, rate of T2D complications**  **(95 % CI)** |  |  |  |  |
| Weibull Gamma | 0.857 (0.778 to 0.944) | >0.001 | Normal | 0.857 (0.084) |
| Age Coefficient | -0.062 (-0.075 to -0.049) | >0.001 | Normal | -0.062 (0.063) |
| Gender Coefficient | -0.760 (-1.355 to -0.485) | >0.001 | Normal | -0.760 (0.444) |
| Complication Coefficient | 8.430 (7.290 to 9.570) | >0.001 | Normal | 8.430 (1.166) |
