# Supplementary material for: Pharmacy-based screening to detect persons at elevated risk of type 2 diabetes: a cost-utility analysis
Source: BMC Health Serv Res. 2021 Sep 5;21:916. doi: 10.1186/s12913-021-06948-6 (PMC8418722; doi:10.1186/s12913-021-06948-6)
Supplement: Supplementary file 7 — Additional file 7. Age and gender- specific all-cause mortality data in Finland. Table showing the all-cause mortality data according to Statistics Finland. [file 12913_2021_6948_MOESM7_ESM.docx]

**Additional file 7**. Age and gender- specific all-cause mortality data in Finland [1].

| Age | Male | Female |
| --- | --- | --- |
| 0 | 0.0023 | 0.00187 |
| 1 | 0.00016 | 0.00013 |
| 2 | 0.00022 | 0.00004 |
| 3 | 0.00011 | 0.00007 |
| 4 | 0.0001 | 0.00004 |
| 5 | 0.00016 | 0 |
| 6 | 0.00006 | 0 |
| 7 | 0.00003 | 0 |
| 8 | 0 | 0.00007 |
| 9 | 0.00003 | 0.00006 |
| 10 | 0.00006 | 0.00007 |
| 11 | 0.00006 | 0.00013 |
| 12 | 0 | 0.00007 |
| 13 | 0.00003 | 0.00017 |
| 14 | 0.00026 | 0.00024 |
| 15 | 0.00026 | 0.00021 |
| 16 | 0.00023 | 0.00021 |
| 17 | 0.0004 | 0.00028 |
| 18 | 0.00079 | 0.00024 |
| 19 | 0.00067 | 0.00038 |
| 20 | 0.00068 | 0.00058 |
| 21 | 0.00106 | 0.00047 |
| 22 | 0.00105 | 0.00043 |
| 23 | 0.00075 | 0.00029 |
| 24 | 0.00081 | 0.00036 |
| 25 | 0.00093 | 0.00033 |
| 26 | 0.00102 | 0.00029 |
| 27 | 0.00089 | 0.00032 |
| 28 | 0.00078 | 0.00032 |
| 29 | 0.00089 | 0.00040 |
| 30 | 0.00087 | 0.00049 |
| 31 | 0.00102 | 0.00024 |
| 32 | 0.00122 | 0.00027 |
| 33 | 0.00066 | 0.00056 |
| 34 | 0.0010 | 0.00034 |
| 35 | 0.0010 | 0.00048 |
| 36 | 0.00149 | 0.00050 |
| 37 | 0.00118 | 0.00055 |
| 38 | 0.00161 | 0.00056 |
| 39 | 0.00118 | 0.00065 |
| 40 | 0.00149 | 0.00071 |
| 41 | 0.00165 | 0.00056 |
| 42 | 0.00151 | 0.00088 |
| 43 | 0.00145 | 0.00089 |
| 44 | 0.00222 | 0.00099 |
| 45 | 0.0017 | 0.00091 |
| 46 | 0.00193 | 0.00095 |
| 47 | 0.00208 | 0.00108 |
| 48 | 0.00274 | 0.00120 |
| 49 | 0.00303 | 0.00163 |
| 50 | 0.00294 | 0.00179 |
| 51 | 0.0032 | 0.00165 |
| 52 | 0.00395 | 0.00193 |
| 53 | 0.00373 | 0.00267 |
| 54 | 0.00467 | 0.00217 |
| 55 | 0.00528 | 0.00212 |
| 56 | 0.00578 | 0.00263 |
| 57 | 0.00613 | 0.00262 |
| 58 | 0.00636 | 0.00298 |
| 59 | 0.00749 | 0.00402 |
| 60 | 0.00738 | 0.00408 |
| 61 | 0.00827 | 0.00478 |
| 62 | 0.00973 | 0.00412 |
| 63 | 0.01052 | 0.00515 |
| 64 | 0.01195 | 0.00552 |
| 65 | 0.01286 | 0.00771 |
| 66 | 0.01448 | 0.00768 |
| 67 | 0.01504 | 0.00701 |
| 68 | 0.0164 | 0.00866 |
| 69 | 0.01892 | 0.00902 |
| 70 | 0.02172 | 0.00989 |
| 71 | 0.02196 | 0.01181 |
| 72 | 0.02376 | 0.01229 |
| 73 | 0.02833 | 0.01538 |
| 74 | 0.02555 | 0.01319 |
| 75 | 0.03242 | 0.01857 |
| 76 | 0.03525 | 0.01930 |
| 77 | 0.03863 | 0.02226 |
| 78 | 0.04023 | 0.02139 |
| 79 | 0.0492 | 0.03270 |
| 80 | 0.05372 | 0.03274 |
| 81 | 0.05745 | 0.03705 |
| 82 | 0.06701 | 0.04046 |
| 83 | 0.07188 | 0.05036 |
| 84 | 0.08327 | 0.05407 |
| 85 | 0.09174 | 0.06429 |
| 86 | 0.10739 | 0.07453 |
| 87 | 0.12056 | 0.08920 |
| 88 | 0.13193 | 0.09839 |
| 89 | 0.15499 | 0.11483 |
| 90 | 0.17044 | 0.13357 |
| 91 | 0.19946 | 0.14852 |
| 92 | 0.21325 | 0.17531 |
| 93 | 0.23923 | 0.19059 |
| 94 | 0.25818 | 0.20511 |
| 95 | 0.27958 | 0.22908 |
| 96 | 0.290 | 0.25352 |
| 97 | 0.35622 | 0.27094 |
| 98 | 0.36277 | 0.31465 |
| 99 | 0.40181 | 0.32106 |

1. Statistics Finland Official Statistics of Finland (OSF): Deaths [e-publication]. Available online: http://www.stat.fi/til/kuol/meta_en.html (accessed on May 18, 2021).
