# Supplementary material for: Pharmacy-based screening to detect persons at elevated risk of type 2 diabetes: a cost-utility analysis
Source: BMC Health Serv Res. 2021 Sep 5;21:916. doi: 10.1186/s12913-021-06948-6 (PMC8418722; doi:10.1186/s12913-021-06948-6)
Supplement: Supplementary file 8 — Additional file 8. Baseline characteristics of the cohorts used in the Markov model. Table showing the characteristics of the population cohorts by gender. [file 12913_2021_6948_MOESM8_ESM.docx]

**Additional file 8.** Baseline characteristics of the cohorts used in the Markov model.

| **Gender** | **Men** | **Women** | **Both** |
| --- | --- | --- | --- |
| **Population (not excl. T2D)**  **(% of population) (30-79 years)** [1] | 1 673 290 | 1 702 260 | 3 375 550 |
| **Pharmacy visitors (not excl. T2D)**  **(30-79 years)** [2] | 950 664 | 1 119 348 | 2 070 012 |
| **Proportion of** **T2D in whole population**  **(HbA1c ≥ 48 or fasting glucose ≥ 7) (%)** [3] | 14.6 | 9.4 | 11.4 |
| **Pharmacy visitors**  **(excl. T2D)** | 811 867 | 1 014 129 | 1 825 996 |
| **Number of participants who are contacted**  **(9.9% of non-T2D visitors)** | 80 375 | 100 399 | 180 774 |
| **Average age**  **(StopDia all participants, n=5 882)** | 55.1 | 53.0 | 53.5 |

1. Official Statistics of Finland (OSF): Population structure [e-publication]. ISSN=1797-5395. Helsinki: Statistics Finland. 2018. http://www.stat.fi/til/vaerak/index_en.html. Accessed 2 May 2019.

2. The Finnish Medicines Agency and the Social Insurance Institution of Finland. Finnish Statistics on Medicines. Helsinki; 2017.

3. Koponen P, Borodulin K, Lundqvist A, Sääksjärvi K, Koskinen S. Health, functioning and welfare in Finland. The FinHealth 2017 study [in Finnish]. Terveys, toimintakyky ja hyvinvointi Suomessa FinTerveys 2017-tutkimus. Helsinki; 2018. http://urn.fi/URN:ISBN:978-952-343-105-8.
