# Supplementary material for: Pharmacy-based screening to detect persons at elevated risk of type 2 diabetes: a cost-utility analysis
Source: BMC Health Serv Res. 2021 Sep 5;21:916. doi: 10.1186/s12913-021-06948-6 (PMC8418722; doi:10.1186/s12913-021-06948-6)
Supplement: Supplementary file 9 — Additional file 9. The correlations between the Weibull regression coefficients. Table showing the correlations between the coefficients for the Weibull to estimate the risk of T2D and the rate of complications. [file 12913_2021_6948_MOESM9_ESM.docx]

**Additional file 9.** The correlations between the Weibull regression coefficients. FINDRISC 0-6 serves as the reference category.

| **Weibull regression coefficients, risk of T2D** |  |  |  |  |  |  |  |  |
| --- | --- | --- | --- | --- | --- | --- | --- | --- |
|  | **FINDRISC 7-11** | **FINDRISC 12-14** | **FINDRISC 15-19** | **FINDRISC 20+** | **Age coefficient** | **Gender coefficient** | **Constant**  **term** |  |
| **FINDRISC 7-11** | 1 |  |  |  |  |  |  |  |
| **FINDRISC 12-14** | 0.838 | 1 |  |  |  |  |  |  |
| **FINDRISC 15-19** | 0.837 | 0.885 | 1 |  |  |  |  |  |
| **FINDRISC 20+** | 0.763 | 0.829 | 0.873 | 1 |  |  |  |  |
| **Age coefficient** | -0.017 | -0.033 | -0.039 | -0.047 | 1 |  |  |  |
| **Gender coefficient** | 0.042 | 0.058 | 0.125 | 0.213 | -0.011 | 1 |  |  |
| **Constant term** | -0.476 | -0.527 | -0.566 | -0.562 | -0.752 | -0.21 | 1 |  |
| **Weibull regression coefficients, rate of T2D complications** | |  |  |  |  |  |  |  |
|  | | **Age Coefficient** | **Gender Coefficient** | **Constant term** |  |  |  |  |
| **Age Coefficient** | | 1 |  |  |  |  |  |  |
| **Gender Coefficient** | | 0.284 | 1 |  |  |  |  |  |
| **Constant term** | | -0.911 | -0.630 | 1 |  |  |  |  |
